# Poly[[μ-(1-ammonio­ethane-1,1-di­yl)bis­(hydrogenphospho­nato)]diaquachloridodisodium]: a powder X-ray diffraction study

**DOI:** 10.1107/S1600536812018077

**Published:** 2012-04-28

**Authors:** Mwaffak Rukiah, Thaer Assaad

**Affiliations:** a Department of Chemistry, Atomic Energy Commission of Syria (AECS), PO Box 6091, Damascus, Syrian Arab Republic

## Abstract

The title compound, [Na_2_(C_2_H_8_NO_6_P_2_)Cl(H_2_O)_2_]_*n*_, has a polymeric two-dimensional structure extending parallel to (001). The asymmetric unit contains two Na^+^ cations located on a centre of symmetry and on a mirror plane, respectively, one half of a bis-phospho­nate anion (the entire anion is completed by mirror symmetry), one chloride anion on a mirror plane and one water mol­ecule in general positions. The two Na^+^ cations exhibit distorted octa­hedral NaCl_2_O_4_ coordination polyhedra, each consisting of two deprotonated O atoms of the bis-phospho­nate anion, of two water mol­ecules and of two chloride anions. Strong O—H⋯O hydrogen bonds between the –OH group and one of the free O atoms of the bis-phospho­nate anion connect adjacent layers along [100], supported by N—H⋯Cl inter­actions. Intra­layer O—H⋯O and N—H⋯O hydrogen bonds are also observed.

## Related literature

For general background to the use of organic diphospho­nic acids as chelating agents in metal extraction and as drugs to prevent calcification and to inhibit bone resorption, see: Matczak-Jon & Videnova-Adrabinska (2005 ▶); Tromelin *et al.* (1986 ▶); Szabo *et al.* (2002 ▶). For related structures, see: Bon *et al.* (2008 ▶); Maltezou *et al.* (2010 ▶). For standard bond lengths, see: Allen *et al.* (1987 ▶). For background and details of methods applied in data collection and Rietveld refinement, see: Thompson *et al.* (1987 ▶); Finger *et al.* (1994 ▶); Stephens (1999 ▶); Von Dreele (1997 ▶); Boultif & Louër (2004 ▶); Rodriguez-Carvajal (2001 ▶); Roisnel & Rodriguez-Carvajal (2001 ▶); Toby (2001 ▶). For the Le Bail method, see: Le Bail *et al.* (1988 ▶).
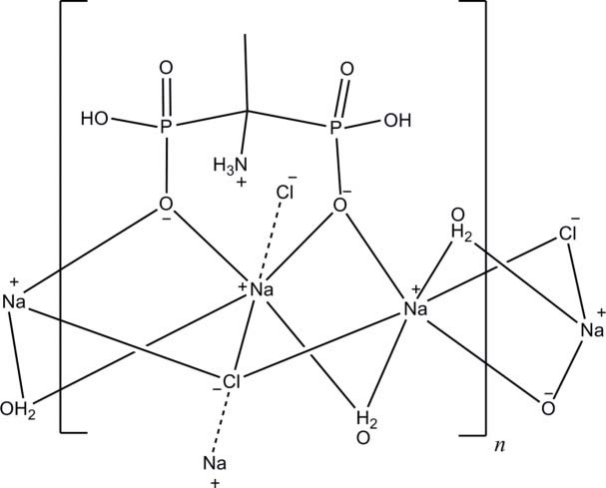


## Experimental

### 

#### Crystal data

[Na_2_(C_2_H_8_NO_6_P_2_)Cl(H_2_O)_2_]
*M* *_r_* = 321.50
Monoclinic, 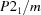

*a* = 5.53806 (4) Å
*b* = 10.50365 (8) Å
*c* = 10.2096 (1) Å
β = 104.0764 (7)°
*V* = 576.06 (1) Å^3^
*Z* = 2
Cu *K*α_1_ radiation
λ = 1.5406 Å
μ = 6.62 mm^−1^
*T* = 298 K
Flat sheet, 8 × 8 mm

#### Data collection

STOE Transmission STADI P diffractometer
Specimen mounting: powder loaded between two Mylar foils
Data collection mode: transmission
Scan method: step
Absorption correction: for a cylinder mounted on the ϕ axis Absorption/surface roughness correction: function number 4 in *GSAS* (Larson & Von Dreele, 2004 ▶). Flat plate transmission absorption correction, terms = 0.51550 0.0000, correction is not refined. *T*_min_ = 0.318, *T*_max_ = 0.451
2θ_min_ = 7.00°, 2θ_max_ = 91.98°, 2θ_step_ = 0.02°

#### Refinement

*R*_p_ = 0.029
*R*_wp_ = 0.038
*R*_exp_ = 0.029
*R*(*F* ^2^) = 0.0257
χ^2^ = 1.769
4250 data points
109 parameters
10 restraints
H atoms treated by a mixture of independent and constrained refinement

### 

Data collection: *WinXPOW* (Stoe & Cie, 1999 ▶); cell refinement: *GSAS* (Larson & Von Dreele, 2004 ▶); data reduction: *WinXPOW*; program(s) used to solve structure: *EXPO2009* (Altomare *et al.*, 2009 ▶); program(s) used to refine structure: *GSAS*; molecular graphics: *ORTEP-3* (Farrugia, 1997 ▶); software used to prepare material for publication: *publCIF* (Westrip, 2010 ▶).

## Supplementary Material

Crystal structure: contains datablock(s) global, I. DOI: 10.1107/S1600536812018077/wm2620sup1.cif

Rietveld powder data: contains datablock(s) I. DOI: 10.1107/S1600536812018077/wm2620Isup2.rtv

Additional supplementary materials:  crystallographic information; 3D view; checkCIF report

## Figures and Tables

**Table 1:** Hydrogen-bond geometry (Å, °)

*D*—H⋯*A*	*D*—H	H⋯*A*	*D*⋯*A*	*D*—H⋯*A*
O3—H3⋯O2^i^	0.82 (2)	1.74 (2)	2.547 (6)	170 (4)
O1W—H1W⋯O1^ii^	0.82 (3)	2.18 (4)	2.978 (6)	166 (5)
O1W—H2W⋯O3^iii^	0.82 (2)	2.28 (3)	2.942 (6)	138 (3)
N1—H1N1⋯O2^iv^	0.87 (3)	2.02 (4)	2.848 (8)	158 (3)
N1—H2N1⋯Cl1	0.87 (3)	2.34 (1)	3.213 (9)	180 (3)

Symmetry codes: (i) 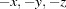; (ii) 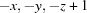; (iii) 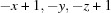; (iv) 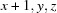.

**Fig. 1.**
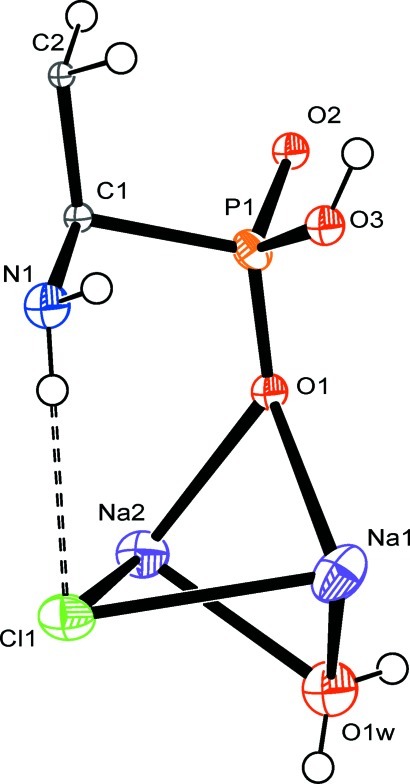
The asymmetric unit of (I) with the atom-labelling scheme. Displacement ellipsoids are drawn at the 50% probability level. H atoms are represented as small spheres of arbitrary radius. Hydrogen bonding is shown as a dashed line.

**Fig. 2.**
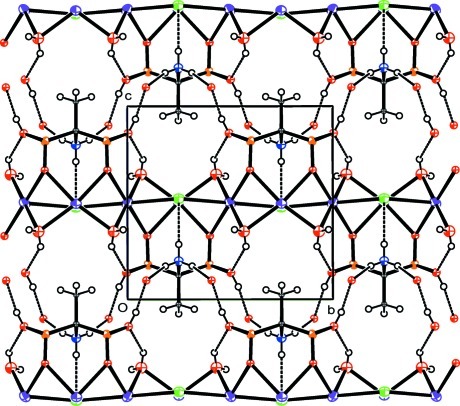
View of crystal packing of (I), showing the formation of the three-dimensional network built from hydrogen bonds (dashed lines).

**Fig. 3.**
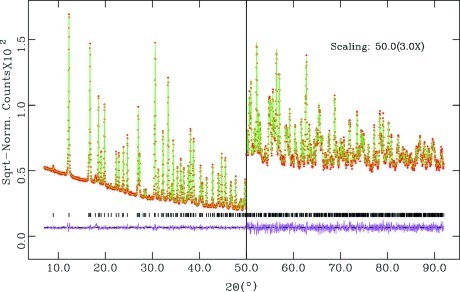
Final Rietveld plot of compound (I). Observed data points are indicated by dots, the best-fit profile (upper trace) and the difference pattern (lower trace) are solid lines. The vertical bars indicate the positions of Bragg peaks.

## supplementary crystallographic information

### Comment

Organic diphosphonic acids are potentially very powerful chelating agents used in metal extractions and have been tested by the pharmaceutical industry for use as efficient drugs preventing calcification and inhibiting bone resorption (Tromelin *et al.*, 1986; Matczak-Jon & Videnova-Adrabinska, 2005). Diphosphonic acids are used in the treatment of Paget disease, osteoporosis and tumoral osteolysis (Szabo *et al.*, 2002). However, it is still not clearly understood why small structural modifications of the bisphosphonates may lead to extensive alterations in their physicochemical, biological and toxicological characteristics (Matczak-Jon & Videnova-Adrabinska, 2005). As a consequence of that, determination of the structure of the bisphosphonates is very important to understand the influence of structural modifications on complex-forming abilities and physiological activities and deriving structure properties relations in general. Structures of the disodium salt of tetrahydrofuranyl-2,2-bisphosphonic acid and of ammonium 1-ammonioethane-1,1-diylbis(hydrogenphosphonate) dihydrate have been reported previously (Maltezou *et al.*, 2010; Bon *et al.*, 2008).

In the present work we report the crystal structure of the sodium salt of 1-ammonioethane-1,1 diyl)bis(hydrogenphosphonate), {[Na_2_(C_2_H_8_NO_6_P_2_)Cl(H_2_O)_2_]}*_n_*, (I). Bond lengths and angles in the anion are comparable with the related structures (Maltezou *et al.*, 2010; Bon *et al.*, 2008) and are in their normal ranges (Allen *et al.*, 1987).

A view of the asymmetric unit of compound (I) is shown in Fig. 1. It contains one half of the anionic bisphosphonate molecule (completed by mirror symmetry), one chloride anion, two Na^+^ cations and one water molecule. The anion is present in a zwitterionic form with two negative charges on the deprotonated O atoms of the phosphonate group and a postive charge on the protonated amino group. The two Na^+^ cations and the chloride anion occupy special positions on an inversion centre for one of the Na cation and on a mirror plane for the other Na^+^ cation and the chloride anion. The two cations exhibit distorted octahedral coordination geometries consisting of two deprotonated O atoms of the bisphosphonate anion and two water molecules in the equatorial plane and two chloride anions in axial positions. The coordination octahedra share faces to make up a linear array directed along [010]. These chains are connected to each other *via* chloride anions to form infinite sheets parallel to (001). The two-dimensional networks are stacked along [001]. The bisphosphonate anions are located above and below the layers, thereby insulating the Na^+^ cations in each layer. The layers are further connected by strong O—H···O hydrogen bonding between adjacent phosphonate groups, supported by N—H···Cl hydrogen bonds (Table 1). N—H···O and two Ow—H···O intralayer hydrogen bonds are also present (Fig. 2, Table 1).

### Experimental

For syntheses of (I), a mixture of acetonitrile (150 ml) and phosphorous acid (16.8 g, 0.2 mol) in acetic acid (10 g, 0.167 mol) was heated at a temperature of 328-338 K and phosphorous trichloride (51.7 g, 0.334 mol) was added slowly under stirring. After completion of the addition, the reaction temperature was raised to 343 -348 K and the reaction continued for 24 h at the same temperature. The reaction mixture was cooled to 333-338 K and water (150 ml) was added slowly at the same temperature. The reaction temperature was then increased to 363-373 K and maintained for the next 4–6 h. The reaction mixture was then cooled to 328-338 K and the reaction mixture pH was adjusted to 4.4–4.8 with sodium hydroxide solution. The reaction mixture was cooled to 278-288 K and the aqueous layer containing the product was separated from the upper acetonitrile layer. The aqueous layer was cooled and maintained at 273-278 K for 3 h. The solid product was separated by filtration and washed with water and finally with methanol to produce the corresponding product, in 77% yield. Appearance: white powder. Melting point about 623 K.

Spectroscopic data of (I): ^1^H-NMR (D_2_O, p.p.m.): *δ* 1.46 (t, 3H, CH_3_, *J*=12.8 Hz). ^13^C{^1^H} NMR (D_2_O, p.p.m.): *δ* 18.2 (1 C; CH_3_), 54.7 (1 C; C– CH_3_). ^31^p{^1^H} NMR (D_2_O, p.p.m.): *δ* 14.53(2P; P—OH). IR (KBr, ν cm^-1^): 3442.2 (NH_2_), 3551.5 (OH), 2393.9 (POH), 1607.6 (O=P—O—H), 1199.5 (P=O). Analytical data for (I): Found: C, 8.00; H, 3.95; N, 4.06; Calculated C, 7.45; H, 4.06; N, 4.34

### Refinement

Except the P, Cl and Na atoms, all other atoms were refined with an isotropic displacement parameter. Several restraints on bonds lengths and angles were applied to H atoms. The H atoms of the NH_3_, OH groups and H atoms of water were located in a difference map. The methyl H atoms were positioned in their idealized geometries using a riding model with C—H = 0.97 Å. The coordinates of these H atoms were restrained to the distances N—H = 0.87 Å, O—H = 0.82 Å and Ow—H = 0.82 Å. All H atoms were refined with isotropic displacement parameters (set to 1.2 times of the *U*_eq_ of the parent atom for methyl H atoms and to 1.5 times of the *U*_eq_ of the parent atom for NH_3_ and OH groups and Ow—H).

The final Rietveld plot of the X-ray diffraction pattern is given in Fig. 3.

### Crystal data

**Table:**

[Na_2_(C_2_H_8_NO_6_P_2_)Cl(H_2_O)_2_]	*D*_x_ = 1.854 Mg m^−^^3^
*M**_r_* = 321.50	Melting point: 623 K
Monoclinic, *P*2_1_/*m*	Cu *K*α_1_ radiation, λ = 1.5406 Å
Hall symbol: -P 2yb	µ = 6.62 mm^−^^1^
*a* = 5.53806 (4) Å	*T* = 298 K
*b* = 10.50365 (8) Å	Particle morphology: Fine powder
*c* = 10.2096 (1) Å	white
β = 104.0764 (7)°	flat sheet, 8 × 8 mm
*V* = 576.06 (1) Å^3^	Specimen preparation: Prepared at 298 K and 101.3 kPa
*Z* = 2	

### Data collection

**Table:**

STOE Transmission STADI P diffractometer	Scan method: step
Radiation source: sealed X-ray tube	Absorption correction: for a cylinder mounted on the φ axis Absorption/surface roughness correction: function number 4 in *GSAS* (Larson & Von Dreele, 2004). Flat plate transmission absorption correction, terms = 0.51550 0.0000, correction is not refined.
Curved Ge(111) monochromator	*T*_min_ = 0.318, *T*_max_ = 0.451
Specimen mounting: powder loaded between two Mylar foils	2θ_min_ = 7.00°, 2θ_max_ = 91.98°, 2θ_step_ = 0.02°
Data collection mode: transmission	

### Refinement

**Table:**

Least-squares matrix: full	Profile function: CW Profile function number 4 with 21 terms Pseudovoigt profile coefficients as parameterized in Thompson *et al.* (1987) Asymmetry correction of Finger *et al.* (1994). Microstrain broadening by Stephens (1999). #1(GU) = 0.000 #2(GV) = 0.000 #3(GW) = 11.529 #4(GP) = 0.000 #5(LX) = 0.000 #6(ptec) = 2.91 #7(trns) = 0.00 #8(shft) = -1.5788 #9(sfec) = 0.00 #10(S/L) = 0.0215 #11(H/L) = 0.0215 #12(eta) = 0.6000 #13(S400 ) = 2.1E-01 #14(S040 ) = 2.3E-02 #15(S004 ) = 1.2E-02 #16(S220 ) = 4.2E-02 #17(S202 ) = 4.6E-02 #18(S022 ) = 1.7E-03 #19(S301 ) = 8.7E-02 #20(S103 ) = -3.8E-05 #21(S121 ) = 2.8E-03 Peak tails are ignored where the intensity is below 0.0010 times the peak Aniso. broadening axis 0.0 0.0 1.0
*R*_p_ = 0.029	109 parameters
*R*_wp_ = 0.038	10 restraints
*R*_exp_ = 0.029	0 constraints
*R*(*F*^2^) = 0.0257	H atoms treated by a mixture of independent and constrained refinement
χ^2^ = 1.769	(Δ/σ)_max_ = 0.08
4250 data points	Background function: GSAS Background function number 1 with 20 terms. Shifted Chebyshev function of 1st kind 1: 914.240 2: -1034.48 3: 577.328 4: -206.578 5: 31.4580 6: 15.1650 7: -17.0889 8: 0.311333 9: 15.3490 10: -12.5113 11: 3.19417 12: 9.78413 13: -11.5493 14: 7.63897 15: -0.448352 16: -4.70971 17: 6.05628 18: -4.89696 19: 6.60474 20: -2.48023
Excluded region(s): none	Preferred orientation correction: March-Dollase AXIS 1 Ratio= 1.12753 h= 0.000 k= 0.000 l= 1.000 Prefered orientation correction range: Min= 0.69761, Max= 1.19727

### Special details

**Table:**

Experimental. All chemical reagents and solvents were of commercial quality and used as received. NMR spectra were recorded on a Bruker Bio spin 400 spectrometer (400 MHz for ^1^H, 100 MHz for ^13^C, 162 MHz for ^31^P). Chemical shifts (δ) were expressed in p.p.m. relative to TMS as an internal standard. IR spectra were recorded on FTIR-JASCO 300E. Melting points were determined using a Stuart SMP3 melting point apparatus. The powder sample of compound (I) was slightly ground in a mortar, loaded into two foils of Mylar and fixed in the sample holder with a mask of suitable internal diameter (8.0 mm). X-ray powder diffraction patterns were obtained on a Stoe Stadi-P diffractometer with monochromatic Cu K_α__1_ radiation (λ = 1.5406 Å) selected using an incident-beam curved-crystal germanium Ge(111) monochromator, using the Stoe transmission geometry (horizontal set-up) with a linear position-sensitive detector (PSD). The pattern was scanned over the angular range 7–92° (2θ)The sample was ground lightly in a mortar, loaded between two Mylar foils and fixed in the sample holder with a mask of 8.0 mm internal diameter.

### Fractional atomic coordinates and isotropic or equivalent isotropic displacement parameters (Å^2^)

**Table:**

	*x*	*y*	*z*	*U*_iso_*/*U*_eq_	
P1	0.1295 (3)	0.10112 (14)	0.1805 (2)	0.01656	
Cl1	0.6926 (3)	0.25	0.5220 (2)	0.02613	
Na1	0.5	0.0	0.5	0.03054	
Na2	0.1724 (5)	0.25	0.5042 (3)	0.02699	
O1	0.1724 (6)	0.0974 (3)	0.3308 (4)	0.0137 (11)*	
O2	−0.1356 (6)	0.0911 (3)	0.0983 (4)	0.0137 (11)*	
O3	0.2987 (8)	−0.0041 (4)	0.1397 (4)	0.0164 (11)*	
O1w	0.2545 (8)	0.0669 (4)	0.6478 (5)	0.0326 (13)*	
N1	0.5321 (15)	0.25	0.1977 (9)	0.022 (2)*	
C1	0.2590 (13)	0.25	0.1280 (8)	0.009 (3)*	
C2	0.2359 (11)	0.25	−0.0250 (6)	0.007 (2)*	
H1c2	0.0613 (15)	0.25	−0.0726 (10)	0.009 (3)*	
H2c2	0.315 (2)	0.1743 (6)	−0.0494 (10)	0.009 (3)*	
H1n1	0.608 (7)	0.185 (3)	0.173 (5)	0.033 (3)*	
H2n1	0.576 (11)	0.25	0.2855 (10)	0.033 (3)*	
H3	0.252 (8)	−0.040 (3)	0.0666 (19)	0.0246 (17)*	
H1w	0.132 (5)	0.021 (4)	0.639 (5)	0.0488 (19)*	
H2w	0.337 (7)	0.077 (4)	0.7255 (17)	0.0488 (19)*	

### Atomic displacement parameters (Å^2^)

**Table:**

	*U*^11^	*U*^22^	*U*^33^	*U*^12^	*U*^13^	*U*^23^
P1	0.0136 (12)	0.0168 (12)	0.0188 (16)	−0.0016 (11)	0.0031 (12)	−0.0026 (13)
Cl1	0.0162 (17)	0.0362 (17)	0.024 (2)	0.0	0.0015 (16)	0.0
Na1	0.037 (3)	0.031 (3)	0.031 (3)	0.008 (2)	0.022 (3)	0.007 (3)
Na2	0.027 (2)	0.030 (2)	0.024 (3)	0.0	0.004 (2)	0.0

### Geometric parameters (Å, º)

**Table:**

P1—O1	1.495 (4)	N1—H2n1	0.870 (14)
P1—O2	1.507 (4)	Na1—Cl1	2.8226 (7)
P1—O3	1.570 (4)	Na2—Cl1^i^	2.707 (3)
P1—C1	1.853 (4)	Na2—Cl1	2.842 (3)
C2—C1	1.537 (9)	Na1—O1	2.408 (3)
C2—H1c2	0.971 (11)	Na1—O1w	2.370 (5)
C2—H2c2	0.969 (9)	Na2—O1	2.389 (4)
N1—C1	1.507 (10)	Na2—O1w	2.394 (5)
N1—H1n1	0.87 (3)		
			
O1—P1—O2	117.4 (2)	Cl1—Na1—O1w	86.43 (10)
O1—P1—O3	107.4 (2)	Cl1—Na1—O1w^iii^	93.57 (10)
O1—P1—C1	110.0 (3)	O1—Na2—O1^ii^	84.3 (2)
O2—P1—O3	111.5 (2)	O1—Na2—O1w	83.14 (14)
O2—P1—C1	106.9 (3)	O1—Na2—O1w^ii^	163.5 (2)
O3—P1—C1	102.7 (3)	O1w—Na2—O1w^ii^	106.9 (3)
P1—C1—P1^ii^	115.1 (4)	Cl1^i^—Na2—Cl1	172.72 (18)
P1—C1—N1	106.1 (4)	Cl1^i^—Na2—O1	103.11 (14)
P1—C1—C2	110.6 (3)	Cl1^i^—Na2—O1^v^	92.54 (2)
N1—C1—C2	107.8 (7)	Cl1^i^—Na2—O1w	90.14 (14)
C1—C2—H1c2	109.6 (4)	Cl1^i^—Na2—O1w^v^	90.02 (3)
C1—C2—H2c2	109.3 (4)	Na1—Cl1—Na1^vi^	136.97 (7)
H1c2—C2—H2c2	109.3 (5)	Na1—Cl1—Na2	68.74 (4)
H2c2—C2—H2c2^ii^	110.1 (11)	Na1—Cl1—Na2^vii^	110.65 (4)
C1—N1—H1n1	111 (4)	Na2—Cl1—Na2^vii^	172.72 (18)
C1—N1—H2n1	119 (4)	P1—O1—Na1	130.6 (2)
H1n1—N1—H1n1^ii^	103 (5)	P1—O1—Na2	135.7 (2)
H1n1—N1—H2n1	105 (4)	Na1—O1—Na2	83.63 (13)
O1—Na1—O1^iii^	180.0	Na1—O1w—Na2	84.34 (16)
O1—Na1—O1w	83.23 (13)	Na1—O1w—H1w	110 (4)
O1—Na1—O1w^iii^	96.77 (13)	Na1—O1w—H2w	113 (4)
O1w—Na1—O1w^iii^	180.0	Na2—O1w—H1w	112 (4)
Cl1—Na1—Cl1^iv^	180.0	Na2—O1w—H2w	118 (3)
Cl1—Na1—O1	82.29 (9)	H1w—O1w—H2w	116 (4)
Cl1—Na1—O1^iii^	97.71 (9)		
			
Na2—Cl1—Na1—O1	43.53 (11)	Cl1—Na1—O1—Na2	−50.23 (10)
Na2—Cl1—Na1—O1W	−40.11 (13)	O1W—Na1—O1—P1	−174.5 (3)
Na1—Cl1—Na2—O1	−43.99 (9)	O1W—Na1—O1—Na2	37.07 (14)
Na1—Cl1—Na2—O1W	39.68 (12)	Cl1^iv^—Na1—O1—P1	−81.8 (2)
O2—P1—O1—Na1	142.2 (2)	O1W^iii^—Na1—O1—P1	5.5 (3)
O2—P1—O1—Na2	−85.9 (3)	O1W^iii^—Na1—O1—Na2	−142.93 (14)
O3—P1—O1—Na1	15.7 (3)	Cl1—Na1—O1W—Na2	45.75 (12)
O3—P1—O1—Na2	147.7 (3)	O1—Na1—O1W—Na2	−36.91 (13)
C1—P1—O1—Na1	−95.3 (3)	O1^iii^—Na1—O1W—Na2	143.09 (13)
C1—P1—O1—Na2	36.7 (4)	Cl1—Na2—O1—P1	−95.6 (3)
O1—P1—C1—N1	58.6 (5)	Cl1—Na2—O1—Na1	49.77 (8)
O1—P1—C1—C2	175.2 (4)	O1W—Na2—O1—P1	178.0 (3)
O1—P1—C1—P1^ii^	−58.5 (5)	O1W—Na2—O1—Na1	−36.65 (14)
O2—P1—C1—N1	−172.9 (4)	Cl1^i^—Na2—O1—P1	89.4 (3)
O2—P1—C1—C2	−56.3 (5)	O1^ii^—Na2—O1—P1	−12.7 (3)
O2—P1—C1—P1^ii^	70.0 (5)	O1^ii^—Na2—O1—Na1	132.66 (13)
O3—P1—C1—N1	−55.5 (5)	Cl1—Na2—O1W—Na1	−45.41 (11)
O3—P1—C1—C2	61.2 (5)	O1—Na2—O1W—Na1	37.27 (14)
O3—P1—C1—P1^ii^	−172.6 (4)	O1W^ii^—Na2—O1W—Na1	−129.37 (17)
Cl1—Na1—O1—P1	98.2 (2)		

Symmetry codes: (i) *x*−1, *y*, *z*; (ii) *x*, −*y*+1/2, *z*; (iii) −*x*+1, −*y*, −*z*+1; (iv) −*x*+1, *y*−1/2, −*z*+1; (v) *x*, −*y*+3/2, *z*; (vi) −*x*+1, *y*+1/2, −*z*+1; (vii) *x*+1, *y*, *z*.

### Hydrogen-bond geometry (Å, º)

**Table:**

*D*—H···*A*	*D*—H	H···*A*	*D*···*A*	*D*—H···*A*
O3—H3···O2^viii^	0.82 (2)	1.74 (2)	2.547 (6)	170 (4)
O1W—H1W···O1^ix^	0.82 (3)	2.18 (4)	2.978 (6)	166 (5)
O1W—H2W···O3^iii^	0.82 (2)	2.28 (3)	2.942 (6)	138 (3)
N1—H1N1···O2^vii^	0.87 (3)	2.02 (4)	2.848 (8)	158 (3)
N1—H2N1···Cl1	0.87 (3)	2.34 (1)	3.213 (9)	180 (3)

Symmetry codes: (iii) −*x*+1, −*y*, −*z*+1; (vii) *x*+1, *y*, *z*; (viii) −*x*, −*y*, −*z*; (ix) −*x*, −*y*, −*z*+1.
